# Supplementary material for: Climate change has increased the odds of extreme regional forest fire years globally
Source: Nat Commun. 2025 Jul 10;16:6390. doi: 10.1038/s41467-025-61608-1 (PMC12246202; doi:10.1038/s41467-025-61608-1)
Supplement: Supplementary file 1 — Supplementary Information [file 41467_2025_61608_MOESM1_ESM.pdf]

Supplementary Materials for

Climate change has increased the odds of extreme regional forest fire years globally

John T. Abatzoglou[1*], Crystal K. Kolden[1], Alison C. Cullen[2], Mojtaba Sadegh[3], Emily L. Williams[4], Marco Turco[5], Matthew W. Jones[6]

[1] School of Engineering, University of California, Merced, USA
[2] Evans School of Public Policy and Governance, University of Washington, Seattle, USA
[3] Department of Civil Engineering, Boise State University, USA
[4] Sierra Nevada Research Institute, University of California, Merced, USA
[5] Department of Physics, University of Murcia, Spain
[6] Tyndall Centre for Climate Change Research, School of Environmental Sciences, University of East Anglia, Norwich, UK.

*Corresponding author. Email: jabatzoglou@ucmerced.edu

This PDF file includes:

Supplementary Text
Figs. S1 to S8
Tables S1 to S5

Supplemental Figures

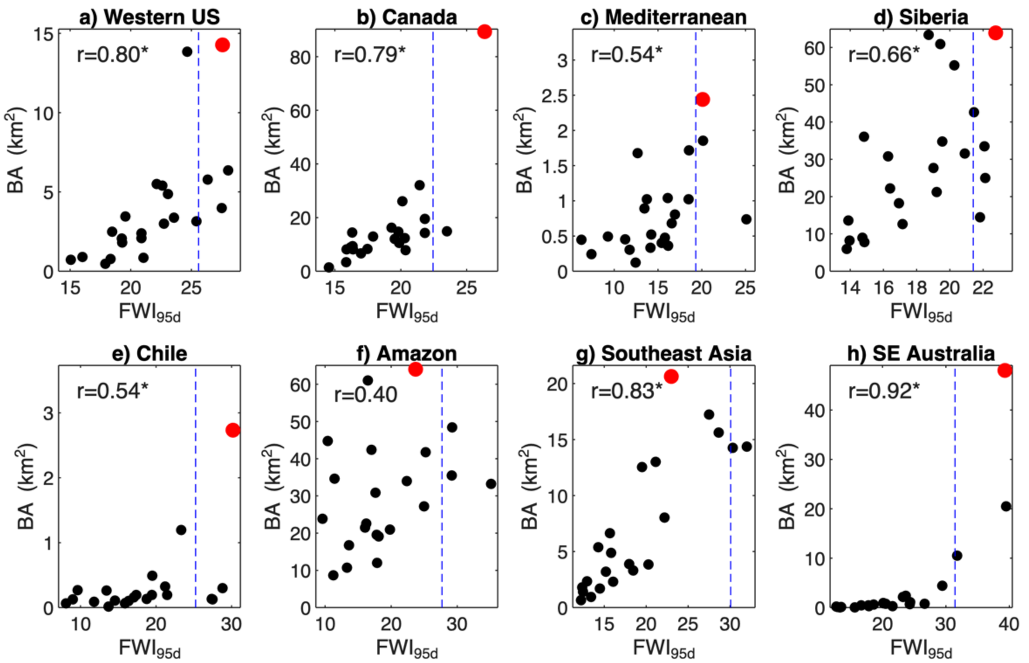

Fig S1: Scatterplot of the number of days with FWI exceeding the 95th percentile (FWI_{95d}) and annual forest burned area for each of the 9 study areas. The red dots denote the largest fire year on record. The r-value is the Pearson' correlation between the logarithm of burned area and FWI_{95d} during 2002–2023 with an asterisk (*) denoting statistical significance ($p<0.05$). The vertical blue-dashed line shows the 1-in-15 year FWI_{95d} value calculated from 1979–2023 data.

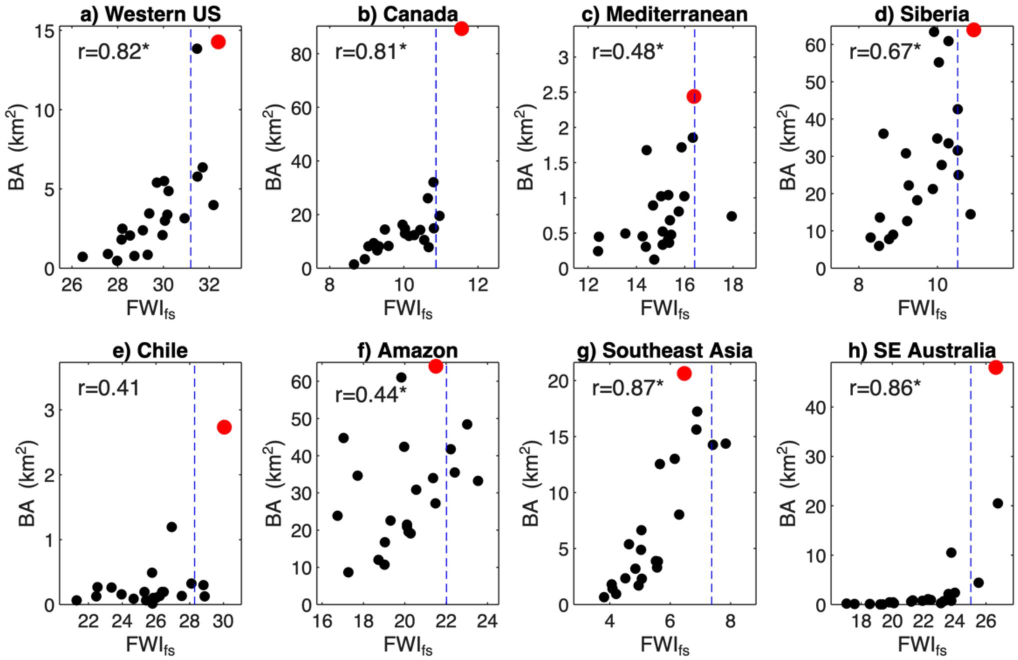

Fig S2: Scatterplot of the maximum 90-day mean FWI (FWI_{fs}) and annual forest burned area for each of the 9 study areas. The red dots denote the largest fire year on record. The r-value is the Pearson' correlation between the logarithm of burned area and FWI_{fs} during 2002–2023 with an asterisk (*) denoting statistical significance ($p<0.05$). The vertical blue-dashed line shows the 1-in-15 year FWI_{fs} value calculated from 1979–2023 data.

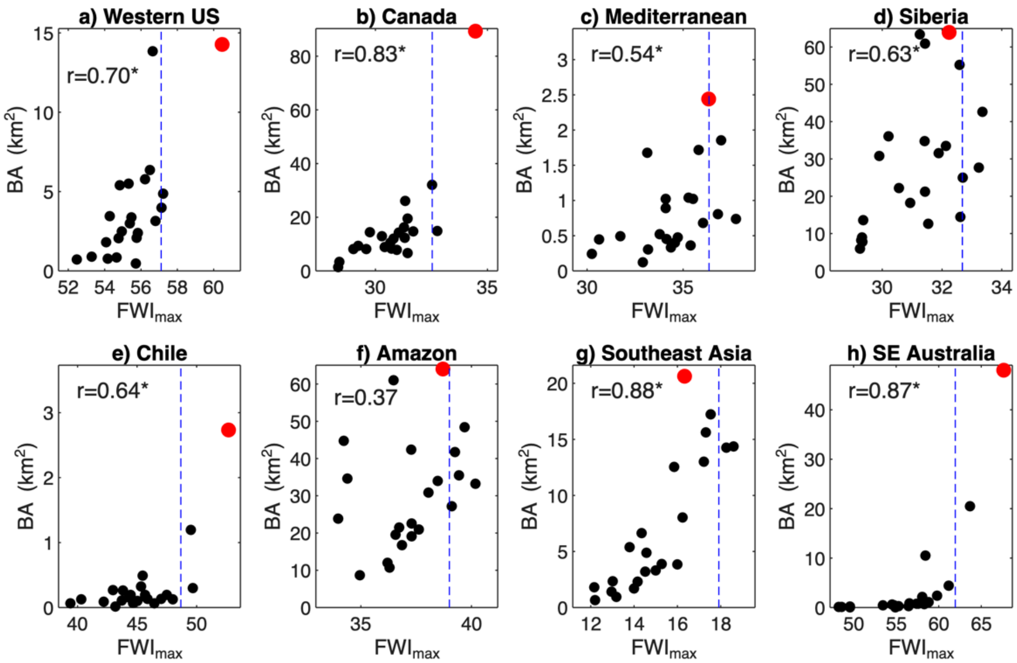

Fig S3: Scatterplot of the annual maximum FWI (FWI_{max}) and annual forest burned area for each of the 9 study areas. The red dots denote the largest fire year on record. The r-value is the Pearson' correlation between the logarithm of burned area and FWI_{max} during 2002–2023 with an asterisk (*) denoting statistical significance ($p<0.05$). The vertical blue-dashed line shows the 1-in-15 year FWI_{max} value calculated from 1979–2023 data.

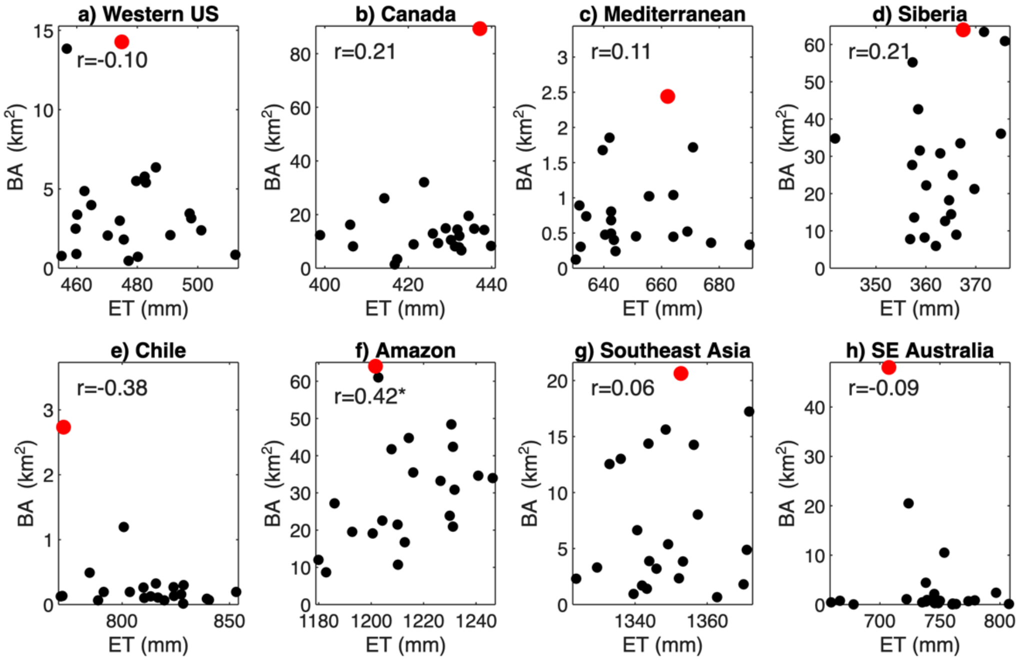

Fig S4: Scatterplot of previous fire year evapotranspiration (ET) and annual forest burned area for each of the 9 study areas. The red dots denote the largest fire year on record. The r-value is the Pearson' correlation between the logarithm of burned area and ET during 2002–2023 with an asterisk (*) denoting statistical significance ($p<0.05$).

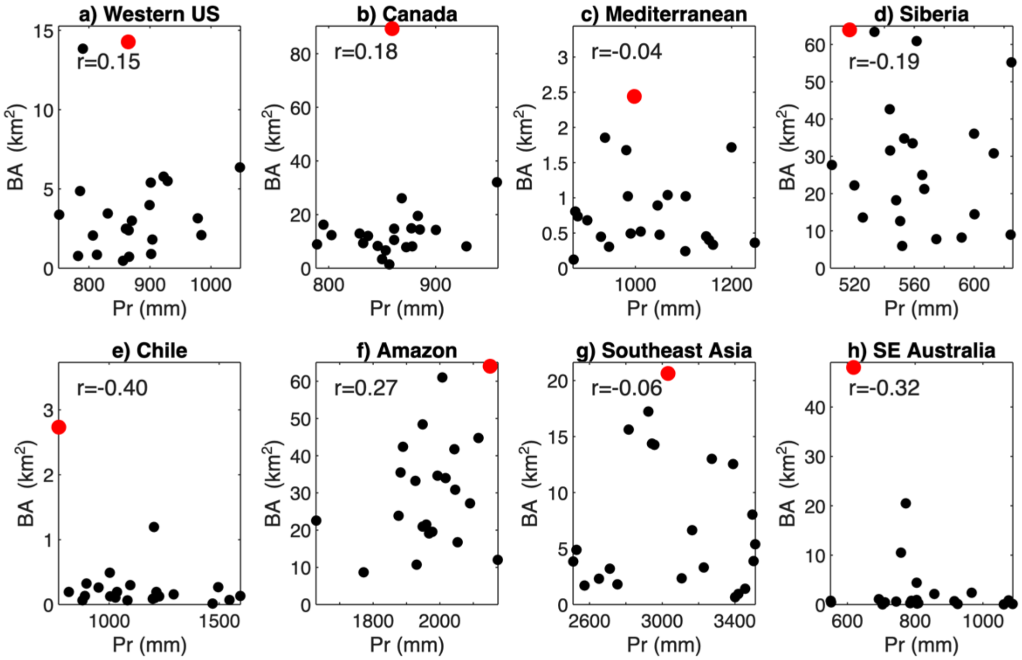

Fig S5: Scatterplot of previous fire year precipitation (Pr) and annual forest burned area for each of the 9 study areas. The red dots denote the largest fire year on record. The r-value is the Pearson' correlation between the logarithm of burned area and Pr during 2002–2023 with an asterisk (*) denoting statistical significance ($p<0.05$).

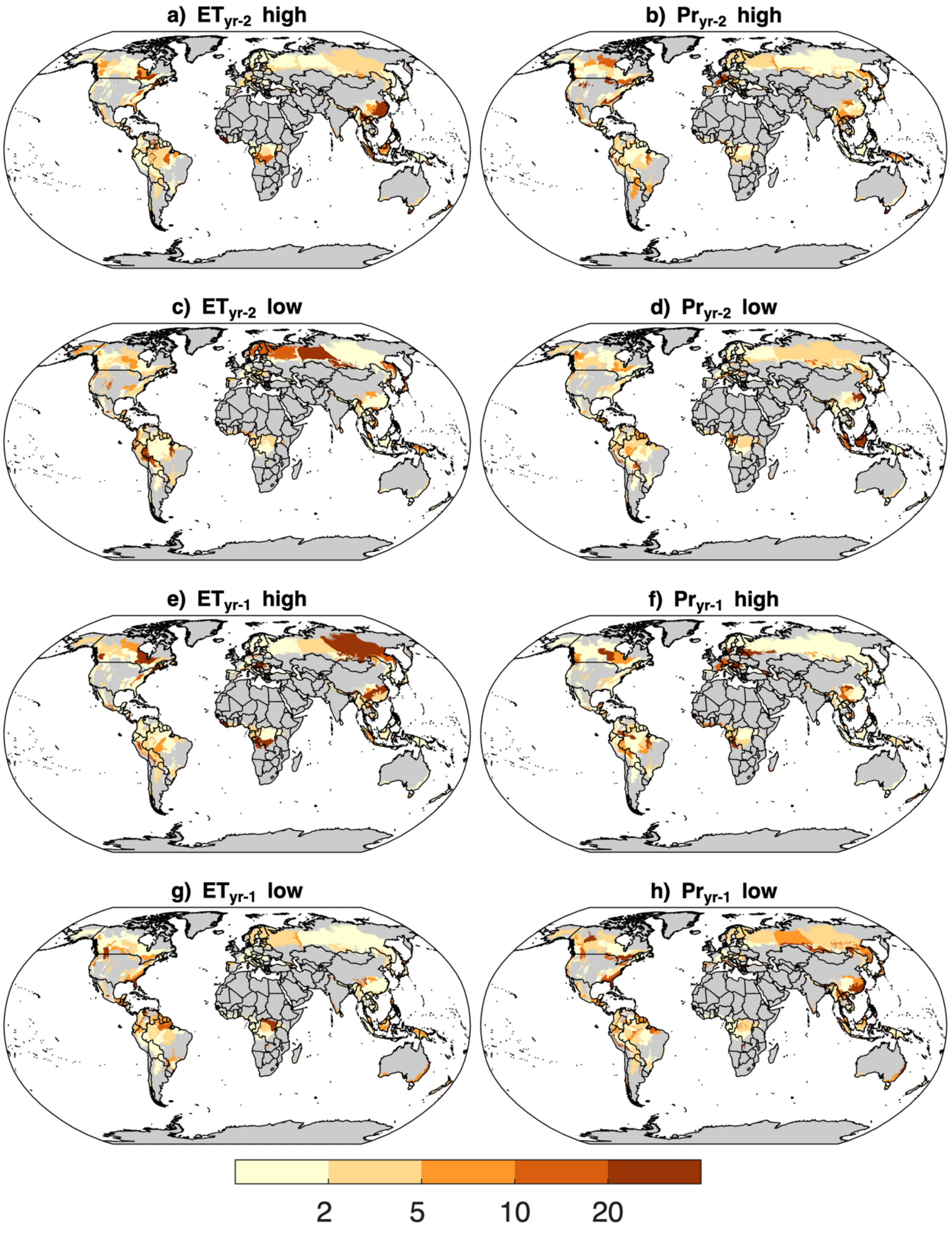

Fig S6: Estimates of the return period of antecedent evapotranspiration (ET) and precipitation (Pr) for (a-d) two years and (e-h) one year prior (bottom two rows for the largest fire year during the 2002–2023 period in each ecoregion. Panels (a,b,e,f) show results for high values while panels (c,d,g,h) show results for low values. Statistics cover the period 1979–2023. Ecoregions with <20% forest land are excluded and shaded grey.

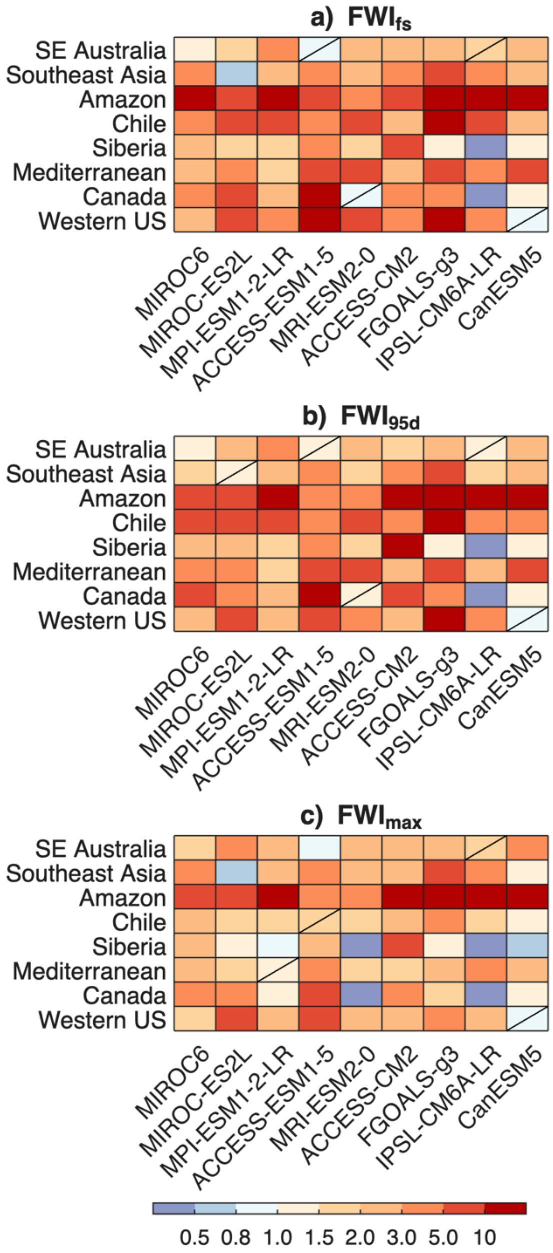

Fig S7: Risk ratio of regional Fire Weather Index (FWI) extremes between *contemporary* (2011–2040) and *quasi pre-industrial* (1851-2000) climates for the case study regions for each GCM. Results are shown for (a) FWI_{fs} – maximum 90 day moving mean FWI, (b) FWI_{95d} – number of days of 95th percentile FWI, and (c) FWI_{max} – annual maximum daily FWI. Results show the risk ratio for each model calculated using pooled ensemble members for the contemporary and quasi pre-industrial periods. Non-significant changes in risk ratio (where the 95% confidence interval includes 1) are denoted by a diagonal hash mark. Lastly, models are ordered from left to right based on the change in annual global mean temperature between 2011-2040 and the quasi-preindustrial period.

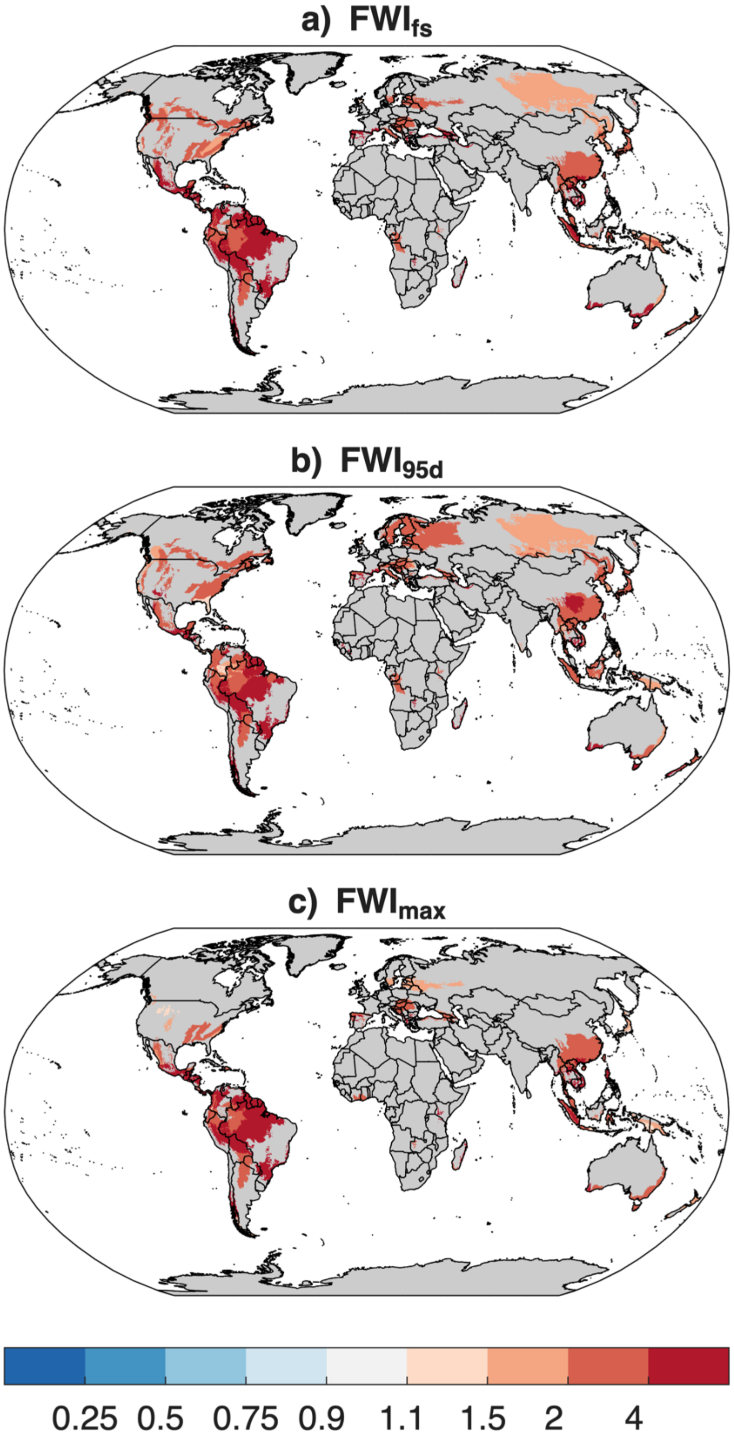

Fig S8: As in Figure 5 showing multi-model median changes in risk ratio for Fire Weather Index (FWI) extremes between the contemporary (2011-2040) period and quasi pre-industrial (1851-1900) period but isolated to forested ecoregions where at least 7 of the 9 models agree on the sign of change. Results are shown for (a) FWI_{fs} – maximum 90 day moving mean FWI, (b) FWI_{95d} – number of days of 95th percentile FWI, and (c) FWI_{max} – annual maximum daily FWI.

	Median RR	RR≥2	RR<1
FWI_{fs}	2.43 (1.92-3.04)	65% (49-75%)	3% (3-38%)
FWI_{95d}	2.52 (1.61-3.20)	63% (46-81%)	2% (3-37%)
FWI_{max}	1.88 (1.61-2.98)	46% (44-69%)	8% (4-39%)

Table S1: Median risk ratio (RR) of the 15-year return interval of maximum 90-day mean FWI defined based on the historical record (1979-2023) between the contemporary (2011-2040) period and the quasi-preindustrial (1851-1900) period. The third and fourth columns show the fraction of global forested land when the nine climate model median change was least double during the contemporary (2011-2040) period compared with the quasi-preindustrial (1851-1900) period (risk ratio, RR ≥2) and was less than the pre-industrial (risk ratio <1), respectively. The range of changes for individual models are shown in parentheses. Note that the multi-model median changes reported here are calculated by first taking the median change for each model separately by each ecoregion, after which the global statistics across forested land are calculated and reported. Results are shown for the three Fire Weather Index (FWI) extremes: FWI_{fs} – maximum 90 day moving mean FWI, FWI_{95d} – number of days of 95th percentile FWI, and FWI_{max} – annual maximum daily FWI.

Model Name	Median RR	RR>2 (%)	RR<1 (%)
ACCESS-ESM1-5	3.04	71	7.7
ACCESS-CM2	2.77	64.3	12.3
FGOALS-g3	2.57	56.2	16.3
CanESM5	1.92	48.8	24
MRI-ESM2-0	2.31	55.4	16.9
MPI-ESM1-2-LR	2.47	56.6	12.9
MIROC6	2.78	74.5	2.6
MIROC-ES2L	2.04	50	14.3
IPSL-CM6A-LR	2.11	51.4	38

Table S2: Risk ratio (RR) of the 15-year return interval of maximum 90-day mean FWI defined based on the historical record (1979-2023) between the contemporary (2011-2040) period and the quasi-preindustrial (1851-1900) period. Reported are the median RR across global forested lands, the percent of lands where RR ≥2, and the percent of lands where RR<1.

**Model Name**	**Median RR**	**RR>2 (%)**	**RR<1 (%)**
ACCESS-ESM1-5	2.94	2.08	71
ACCESS-CM2	3.2	2.01	64.3
FGOALS-g3	2.53	1.86	56.2
CanESM5	1.89	1.86	48.8
MRI-ESM2-0	2.37	1.79	55.4
MPI-ESM1-2-LR	2.68	1.98	56.6
MIROC6	2.88	2.98	74.5
MIROC-ES2L	2.23	1.62	50
IPSL-CM6A-LR	1.61	1.61	51.4

Table S3: Risk ratio (RR) of the 15-year return interval of FWI exceeding the 95th percentile defined based on the historical record (1979-2023) between the contemporary (2011-2040) period and the quasi-preindustrial (1851-1900) period. Reported are the median RR across global forested lands, the percent of lands where RR ≥2, and the percent of lands where RR<1.

**Model Name**	**Median RR**	**RR>2 (%)**	**RR<1 (%)**
ACCESS-ESM1-5	2.08	71	70
ACCESS-CM2	2.01	64.3	67.1
FGOALS-g3	1.86	56.2	57
CanESM5	1.86	48.8	45.8
MRI-ESM2-0	1.79	55.4	56.8
MPI-ESM1-2-LR	1.98	56.6	58.9
MIROC6	2.98	74.5	81.4
MIROC-ES2L	1.62	50	62.1
IPSL-CM6A-LR	1.61	51.4	46.3

Table S4: Risk ratio (RR) of the 15-year return interval of annual maximum FWI defined based on the historical record (1979-2023) between the contemporary (2011-2040) period and the quasi-preindustrial (1851-1900) period. Reported are the median RR across global forested lands, the percent of lands where RR $\geq$2, and the percent of lands where RR<1.

**Model Name**	**# Ensembles**	**Climate Sensitivity**	**GMT (Δ°C)**
ACCESS-ESM1-5	40	3.88	1.33
ACCESS-CM2	5	4.66	1.38
FGOALS-g3	3	2.87	1.46
CanESM5	50	5.64	2.20
MRI-ESM2-0	5	3.13	1.34
MPI-ESM1-2-LR	30	3.03	1.29
MIROC6	33	2.60	1.04
MIROC-ES2L	30	2.66	1.14
IPSL-CM6A-LR	11	4.70	1.70

Table S5: CMIP6 models used, number of ensemble members considered, equilibrium climate sensitivity, and calculated difference in global mean temperature between 2011-2040 (SSP2-4.5) versus 1851-1900.
